# CRISPR/Cas12a Mediated Genome Editing Enhances *Bombyx mori* Resistance to BmNPV

**DOI:** 10.3389/fbioe.2020.00841

**Published:** 2020-07-15

**Authors:** Zhanqi Dong, Qi Qin, Zhigang Hu, Xinling Zhang, Jianghao Miao, Liang Huang, Peng Chen, Cheng Lu, Minhui Pan

**Affiliations:** ^1^State Key Laboratory of Silkworm Genome Biology, Southwest University, Chongqing, China; ^2^Key Laboratory of Sericultural Biology and Genetic Breeding, Ministry of Agriculture and Rural Affairs, Southwest University, Chongqing, China

**Keywords:** CRISPR/Cas12a, genome editing, antiviral therapy, *Bombyx mori*, BmNPV

## Abstract

CRISPR/Cas12a (Cpf1) is a single RNA-guided endonuclease that provides new opportunities for targeted genome engineering through the CRISPR/Cas9 system. Only AsCas12a has been developed for insect genome editing, and the novel Cas12a orthologs nucleases and editing efficiency require more study on insects. We compared three Cas12a orthologs nucleases, AsCas12a, FnCas12a, and LbCas12a, for their editing efficiencies and antiviral abilities. The three Cas12a efficiently edited the *Bombyx mori* nucleopolyhedrovirus (BmNPV) genome and inhibited BmNPV replication in BmN-SWU1 cells. The antiviral ability of the FnCas12a system was more efficient than that of the SpCas9 system after infection by BmNPV. We created FnCas12a × gIE1 and SpCas9 × sgIE1 transgenic hybrid lines and evaluated the gene-editing efficiency of different systems at the same target site. We improved the antiviral ability using the FnCas12a system in transgenic silkworm. This study demonstrated the use of the CRISPR/Cas12a system to achieve high editing efficiencies, and increase disease resistance in the silkworm.

## Introduction

Genome editing introduces DNA mutations in the form of insertions, deletions or base substitutions within selected DNA sequences (Backes et al., 2018). Clustered regularly interspaced short palindromic repeats (CRISPR) gene-editing technology has been used in gene function research, genetic improvement, modeling biology and gene therapy (Burgess, 2013; Alves-Bezerra et al., 2019; Beretta and Mouquet, 2019; Li et al., 2019). Three effector proteins of class 2 type V CRISPR systems, the CRISPR/CRISPR-associated 12a (Cas12a, known as Cpf1) proteins of *Lachnospiraceae bacterium* (LbCas12a), *Francisella novicida* (FnCas12a), and *Acidaminococcus* sp. (AsCas12a), have been shown to efficiently edit mammalian cell genomes with more efficient genome editing than the widely used *Streptococcus pyogenes* CRISPR/CRISPR-associated 9 (SpCas9) (Fagerlund et al., 2015; Zetsche et al., 2015; Gao et al., 2017). However, the CRISPR/Cas12a system has rarely been used for insect genome editing research and antiviral therapy (Ma et al., 2017).

The silk industry suffers great economic losses due to *B. mori* nucleopolyhedrovirus (BmNPV) infection (Isobe et al., 2004; Subbaiah et al., 2013; Dong et al., 2018a). CRISPR genome editing is an efficient and widely used technology for anti-BmNPV gene therapy, viral gene function research, and screening of potential targets in BmNPV infection (Dong et al., 2018a, b; Dong et al., 2018b). We first reported on a highly efficient virus-inducible gene-editing system, which demonstrated that CRISPR/Cas9 could edit the BmNPV genome and effectively inhibit virus proliferation (Dong Z.Q. et al., 2016). Chen et al. (2017) effectively inhibited BmNPV proliferation and replication by editing the *ie-1* and *me53* of BmNPV immediate early genes in transgenic silkworm. We improved the antiviral ability of transgenic silkworm nearly 1,000-fold by editing the two target sites of the *ie-1* gene to produce a large fragment deletion (Dong et al., 2018a). The CRISPR/Cas9 gene-editing technology has also been used in antiviral resistance breeding by editing host factors and viral key genes in BmNPV infection. However, the antiviral resistance level using this system has reached a plateau (Dong et al., 2018b, 2019a,b).

The CRISPR/Cas12a system (Cpf1) is a single RNA-guided endonuclease used for genome editing (Fagerlund et al., 2015). The Cas12a enzyme has several gene-editing characteristics that differ from the Cas9 system (Zetsche et al., 2015; Dong D. et al., 2016). One major difference between the Cas12a and Cas9 systems is that Cas12a recognizes a T-rich protospacer-adjacent motif (PAM), while Cas9 recognizes a G-rich PAM (Fonfara et al., 2016). The Cas12a system increases number of the potential target sites that can be used for CRISPR-mediated gene-editing (Fonfara et al., 2016). Cas12a enzyme requires one U6 (Pol-III) promoter to drive small CRISPR-derived RNA (crRNAs, 42–44-nt per-crRNA, 19-nt repeat and 23–25-nt spacer). However, the Cas9 enzyme requires a single guide RNA (sgRNA) derived from the fusion of CRISPR RNA (crRNA) and *trans*-activating crRNA (tracrRNA) (Burgess, 2013; Fonfara et al., 2016). Multiple crRNAs can be expressed as a single transcript to generate functional individual crRNAs after processing through Cas12a nuclease; this can increase the efficiency of crRNA entry into cells (Fagerlund et al., 2015; Nakade et al., 2017). Cas12a nuclease also generates a 5-bp staggered DNA double-strand break ends that are formed downstream of the PAM sequence, while Cas9 nuclease only formed a blunt-end cut 3 bp upstream of the PAM sequence (Mahfouz, 2017; Nakade et al., 2017). The unique editing features of the Cas12a system are conducive to overcoming the limitations of the Cas9 system.

We investigated the ability of AsCas12a, FnCas12a, and LbCas12a to edit BmNPV genomes in *B. mori*. Our goals were to compare the gene-editing efficiency of the Cas12a and Cas9 systems in anti-BmNPV therapy and to develop transgenic silkworms with BmNPV resistance. Initially, an AsCas12a, FnCas12a and LbCas12a-based gene-editing vector and the crRNA expression cassette were developed. Then, different Cas12a nuclease activities with crRNA derived by the U6 promoter were evaluated for gene-editing efficiency and antiviral ability in BmN-SWU1 cells. The antiviral abilities of FnCas12a and SpCas9 systems, which are widely used for BmNPV genome editing, were compared. Finally, the gene-editing efficiency and resistance level of the transgenic FnCas12a and SpCas9 lines were evaluated by mortality analyses, sequencing and viral gene transcription in transgenic silkworms.

## Results

### CRISPR/Cas12a System Can Edit the BmNPV Genome

**FIGURE 1:**
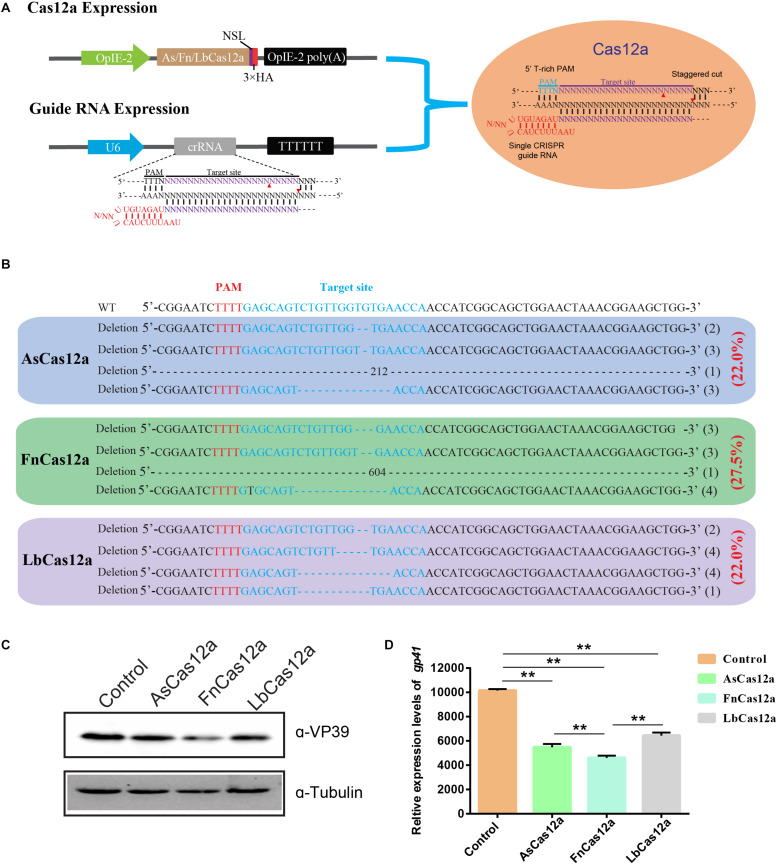
CRISPR/Cas12a system enables BmNPV genome editing. **(A)** Cas12a expression cassette and crRNA target gene site. **(B)** DNA sequencing analysis of high-frequency genome mutations by Cas12a in BmN-SWU1 cells. The BmNPV *ie-1* gene sequence is shown in black on top, the target site of gRNA is in blue, PAM sequence is in red, and the deletion sequence is indicated by dashes. **(C)** Western blot analysis of CRISPR/Cas12a system mediated antiviral activity monitored by the levels of the VP39 (top) and Tubulin (bottom). **(D)** DNA replication analysis of CRISPR/Cas12a system-mediated antiviral activity monitored by the copies of gp41. Error bars represent standard deviations of three biological replicates. ** represents a statistically significant difference at *P* < 0.01.

To determine whether the CRISPR/Cas12a system could be used for gene-editing in silkworm, we examined the functionality of three Cas12a enzymes, AsCas12a, FnCas12a, and LbCas12a, which have been used to edit the genomes of mammal cells. We constructed the expression cassettes of AsCas12a, FnCas12a, and LbCas12a, and connected them with the nuclear localization signal and 3 × HA tag. The expression cassette was initiated by the OpIE2 promoter and terminated by the OpIE2-PA. The crRNA expression cassettes consisting of a 20–21-nt direct repeat and a 23-nt guide sequence were arranged in tandem and driven by a signal U6 promoter of *B. mori*. Then, we transfected BmN-SWU1 cells with individual Cas12a orthologs and gRNA to target endogenous loci containing the 5′ T-rich PAMs (Figure 1A).

Editing the BmNPV *ie-1* gene can effectively inhibit viral replication. We selected the *ie-1* gene as a target for further analysis. To facilitate the quantification and comparison of these nucleases, we constructed one vector system containing Cas12a orthologs and gIE1. After transfecting the Cas12a system in BmN-SWU1 cells, Sanger sequencing analysis revealed that all of the Cas12a enzymes could edit the target site of the *ie-1* gene. To analyze the gene-editing efficiency of different AsCas12a, FnCas12a, and LbCas12a systems, the genome of each group was extracted and T cloning was performed; 20 samples were randomly selected to sequence, and statistical analysis of editing efficiency of the putative cleavage site reached 22.0, 27.5, and 22.0%, respectively (Figure 1B). To further analyze whether the Cas12a system could inhibit virus proliferation, we examined the change in VP39 protein expression after BmNPV infection. Western blot results showed that the expression of VP39 protein was significantly affected by eliminating the viral genome at 48 h post-infection (h p.i.). The VP39 protein expression levels of AsCas12a, FnCas12a, and LbCas12a systems were equivalent to 74.0, 56.0, and 61.0% of the control, respectively (Figure 1C). To demonstrate the antiviral efficiency of the CRISPR/Cas12a system, we also determined the replication of the viral genome through quantitative polymerase chain reaction (qPCR) analysis. The amount of BmNPV DNA was affected after eliminating the viral genome. Compared with the control group, the AsCas12a, FnCas12a, and LbCas12a systems reduced BmNPV DNA by 46.0, 54.6, and 36.5%, respectively (Figure 1D). Statistical analysis also showed that the FnCas12a system also has a significant decrease compared to the AsCas12a and LbCas12a systems (Figure 1D). All of the three constructed CRISPR/Cas12a gene-editing systems significantly inhibited virus replication in *B. mori*, and the FnCas12a system had the greatest antiviral effect.

### Analysis of the Antiviral Ability of CRISPR/Cas9 and CRISPR/Cas12a

**FIGURE 2:**
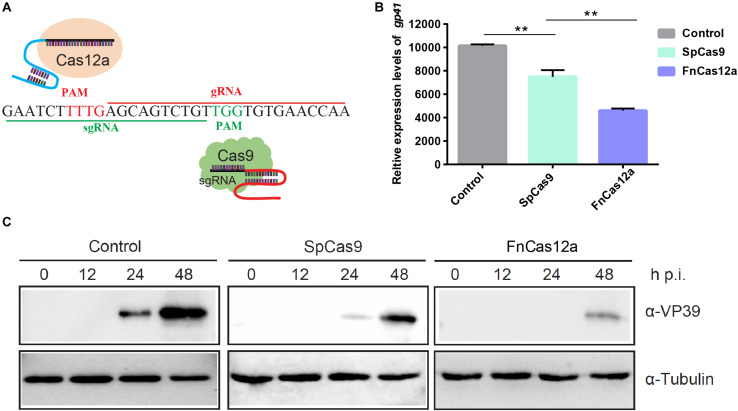
Comparative analysis of the antiviral ability of CRISPR/Cas9 and CRISPR/Cas12a. **(A)** Comparison of CRISPR/Cas9 versus CRISPR/Cas12a-mediated genome editing. Cas12a, Cas9, crRNA, and PAM are shown. **(B)** Analysis of BmNPV DNA replication in CRISPR/Cas12a and CRISPR/Cas9 systems. Error bars represent standard deviations of three biological replicates. ** represents statistically significant differences at *P* < 0.01. **(C)** Western blot analysis of CRISPR/Cas12a and CRISPR/Cas9 system mediated antiviral activity monitored by the levels of the VP39 (top) and Tubulin (bottom). The ratios of different types of mutations.

To evaluate the performance of the different CRISPR systems in *B. mori*, we focused on FnCas12a and SpCas9 gene-editing systems for the same target site (Figure 2A). We initially chose the BmNPV *ie-1* gene as the target. PAM profiling of FnCas12a and SpCas9 is shown in Figure 2A. After transfected with FnCas12a × gIE1 and SpCas9 × sgIE1 in BmN-SWU1 cells, the cells infected with the vA4^prm^-EGFP virus at multiplicity of infection (MOI) of 10. At 48 h p.i., viral DNA replication showed that different gene-editing systems could significantly inhibit BmNPV DNA replication, and the FnCas12a system had a greater inhibition effect compared with the SpCas9 system (Figure 2B). Compared to the control, BmNPV DNA replication levels were reduced by 54.6% in the FnCas12a system and more than 38.5% in the SpCas9 system. We analyzed the changes of VP39 protein expression in the FnCas12a and SpCas9 systems. The Western blot analysis showed that the FnCas12a and SpCas9 systems could significantly inhibit VP39 protein expression. The FnCas12a system only detected the VP39 protein at 48 h p.i. (Figure 2C). After the FnCas12a system was transfected in BmN-SWU1 cells and infected with BmNPV, no significant VP39 protein expression was detected in the FnCas12a system at 0–24 h p.i.; however, a weak VP39 protein band was detected in the SpCas9 system at 24 h p.i. The VP39 protein expression of the FnCas12a system was also lower than that of the SpCas9 system at 48 h p.i. (Figure 2C). These results demonstrated that the antiviral ability of the FnCas12a system was more effective than that of the SpCas9 system for BmNPV at the same target site.

### Gene-Editing Efficiency of CRISPR/Cas9 and CRISPR/Cas12a Systems in Transgenic Silkworm

To compare the genome-editing efficiency of CRISPR/Cas12a and CRISPR/Cas9, we constructed FnCas12a and SpCas9 system transgenic vectors. The vectors pBac[IE2-FnCas12a-OPIE2-PA-3 × P3 EGFP afm], pBac[U6-gIE1-3 × P3 DsRed afm], and pBac[U6-sgIE1-3 × P3 DsRed afm] expressed the FnCas12a protein, gRNA and the sgRNA target sequence, respectively. The SpCas9 transgenic line was studied previously (Dong et al., 2018a).

**FIGURE 3:**
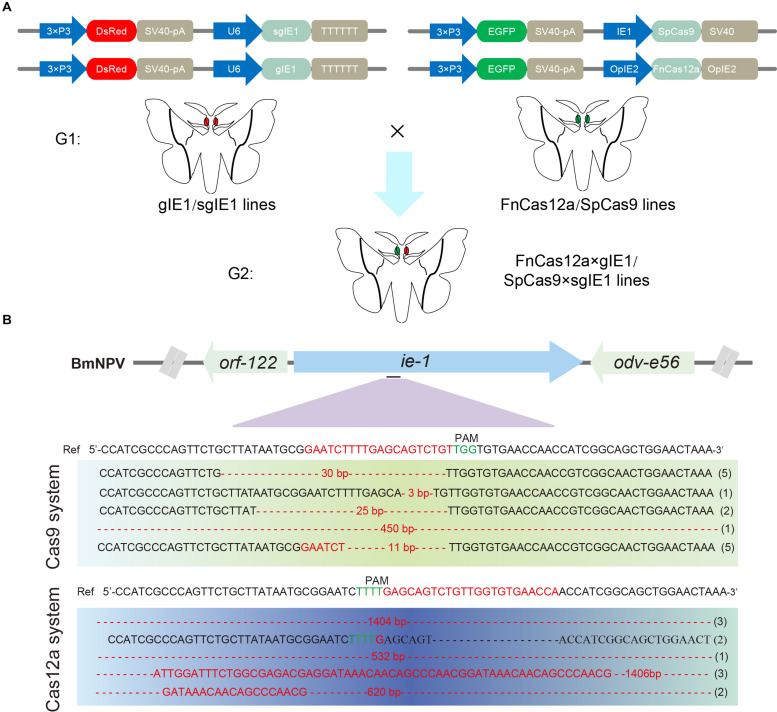
Comparison of gene-editing efficiency of CRISPR/Cas9 and CRISPR/Cas12a systems in transgenic silkworm. **(A)** Schematic presentation of transgenic vector construction of pBac[OpIE2prm-Cas12a-OpIE2-PA-3 × P3 EGFP afm], pBac[U6-gIE1-3 × P3 DsRed afm], and pBac[U6-sgIE1-3 × P3 DsRed afm] (top). Double positive individuals FnCas12a × gIE1 and SpCas9 × sgIE1 obtained were screened by fluorescence microscopy (bottom). **(B)** Sequencing results of two transgenic lines generated by mutagenesis at *ie-1* site.

After selection of the FnCas12a, gIE1, SpCas9, and sgIE1-positive transgenic lines, double-positive transgenic FnCas12a × gIE1 and SpCas9 × sgIE1 lines were obtained through FnCas12a and gIE1 or Cas9 and sgIE1 transgenic line hybridization (Figure 3A). The FnCas12a × gIE1 line expressed both the FnCas12a protein and gIE1 target sequence, and the SpCas9 × sgIE1 line expressed both the SpCas9 protein and sgIE1 target sequence. In the G2 generation, silkworms with both red fluorescent protein and green fluorescent protein expression in their eyes were the double-positive transgenic FnCas12a × gIE1 or SpCas9 × sgIE1 lines (Figure 3A).

To evaluate the potential off-target effects of FnCas12a, we examined all possible off-target sites with high sequence similarity to gIE1 in the silkworm genomes. We selected three non-specific editing sites with the highest similarity for further confirmation by PCR in transgenic lines. Among the three predicted off-targeting sites, we did not detect any off-target mutations in the FnCas12a × gIE1 transgenic lines (Table 1). These results showed that the FnCas12a systems used in antiviral research had no significant effects on non-specific loci even for editing a highly similar site in the silkworm.

**TABLE 1:** Off-target analysis of CRISPR/Cas9 system in the transgenic silkworm.

Target site	Off-target site	Matches sequence	Position	Off-target ratio
SgIE1–360	sgIE1-360: TTTTGAGCAGTCTGTTGGTGTGAACCA	23 + PAM	BmNPV:117354-117377	
	OT1-sgIE1-360: TTAAAGTTTGAGCAGTCTCATGC	9 + PAM	Bomo_Chr1: 14285262-14285272	0/20
	OT1-sgIE1-360: GGAATTTTGAGCAGTCGTTGGT	8 + PAM	Bomo_Chr10: 10751545-10751555	0/19
	OT1-sgIE1-360: TGTAAATTTGAGCAGTCTTACA	9 + PAM	Bomo_Chr14: 175299-175309	0/15

### Silkworm Resistance to BmNPV Conferred by the CRISPR/Cas12a System

To compare the gene-editing efficiency of CRISPR/Cas9 and CRISPR/Cas12a system in transgenic silkworm, we selected the *ie-1* gene of BmNPV as the target gene site. The two systems targeted the same site of *ie-1*. After infection with occlusion bodies (OBs) under the same conditions, we determined the gene-editing efficiency of the target sites in the transgenic hybrid line, FnCas12a × gIE1 or SpCas9 × sgIE1. Sequencing of PCR fragments from these lines demonstrated that both the CRISPR/Cas9 and CRISPR/Cas12a gene-editing systems were able to edit the *ie-1* gene in the BmNPV genome (Figure 3B). We also found that the sequence of SpCas9 × sgIE1 lines was able to edit the target site within the BmNPV genome, which mainly appeared as the absence of 3–30 bp, and only one colony had large deletions in all sequencing (Figure 3B). In contrast, most clones of the transgenic FnCas12a × gIE1 line showed large deletions, ranging from 500 to 1,400 bp. More than 80% of all sequencings were large deletions (Figure 3B).

**FIGURE 4:**
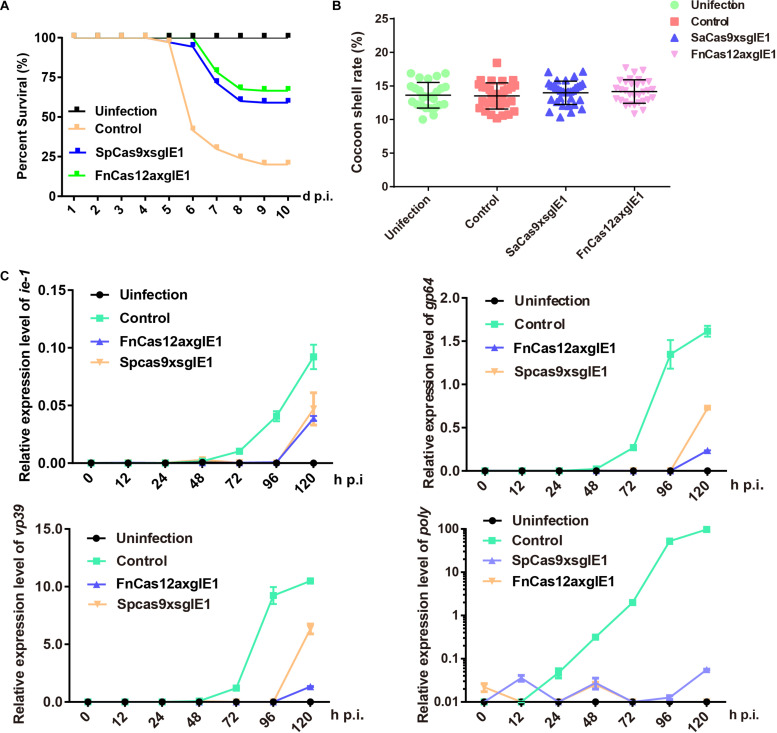
Improved silkworm resistance to virus conferred by the CRISPR/Cas12a system. **(A)** Survival rate of transgenic hybrid FnCas12a × gIE1 and SpCas9 × sgIE1 lines after inoculation with 1 × 10^6^ OBs per 4 fourth instar larva. Each group included 30 larvae and standard deviations of three biological replicates. The mortality was scored at 10 d p.i. **(B)** Cocoon shell rate analysis of the FnCas12a × gIE1 and SpCas9 × sgIE1 lines. Each value represents three biological replicates. **(C)** Gene expression levels of *ie-1*, *gp64*, *vp39*, and *poly* after OB inoculation were analyzed by RT-PCR in two transgenic lines. The data represent of three experiments. ** represents statistically significant differences at the level of *P* < 0.01. NS, not significant.

We determined whether the FnCas12a system could enhance antiviral activity compared with the SpCas9 system in transgenic lines. The transgenic hybrid lines FnCas12a × gIE1, SpCas9 × sgIE1 and the control were infected with 1 × 10^6^ OBs/larva by inoculating 4 fourth instar larvae. Under these conditions, the FnCas12a × gIE1 and SpCas9 × sgIE1 lines significantly reduced the BmNPV infection. The survival rate of the SpCas9 × sgIE1 lines was 59% until 10-day post infection (d p.i.), whereas the control had large-scale mortality after 5–10 d p.i. (Figure 4A). The survival rate of the FnCas12a × gIE1 lines was further increased when they were inoculated with OBs. The FnCas12a × gIE1 lines started to die on 6 d p.i., but the survival rate of the FnCas12a × gIE1 lines was still >65% after 10 d p.i. (Figure 4A). These results suggested that the CRISPR/Cas12a system, in transgenic silkworm, could more effectively improve the antiviral activity (Figure 4A). We determined if the surviving transgenic FnCas12a × gIE1 and SpCas9 × sgIE1 silkworm lines had altered cocoon characteristics after BmNPV infection. We compared the transgenic lines to the control, and found that they were similar to differences ranging from 11 to 18% (Figure 4B).

To compare the antiviral ability of different gene-editing systems, we also analyzed the changes of BmNPV gene expression levels at different stages. Similarly, control, FnCas12a × gIE1 and SpCas9 × sgIE1 transgenic lines inoculating 4 fourth instar larvae were infected with 1 × 10^6^ OBs/larva. At 0, 12, 24, 48, 72, 96, and 120 h p.i., total RNA was isolated from each transgenic line and the samples were analyzed by real time-PCR (RT-PCR). We studied immediate early gene *ie-1*, early gene *gp64*, late gene *vp39* and very late gene *poly* of BmNPV to analyze the viral expression levels at different stages. The RT-PCR results showed that the expression of *ie-1*, *gp64*, *vp39*, and *poly* genes were maintained at a very low level in the FnCas12a × gIE1 and SpCas9 × sgIE1 transgenic lines after BmNPV infection. However, the viral gene expression levels increased as expected in the control (Figure 4C). The viral gene expression levels of FnCas12a × gIE1 and SpCas9 × sgIE1 transgenic lines were 10^4^–10^5^-fold lower compared with the control lines. FnCas12a × gIE1 compared with SpCas9 × gIE1 in different stages was reduced by 10-fold.

## Discussion

Genome editing has the potential to accurately edit the genomes of model organism (Perez-Pinera et al., 2012; Burgess, 2013; Nakade et al., 2017). Cas9, Cas12a- (Cpf1), Cas12b-, Cas13-, Cas3-, and Cas14- based CRISPR systems have been explored for editing human, animals, plants and microbe genomes (Matsoukas, 2018; Schindele et al., 2018; Moon et al., 2019; Morisaka et al., 2019; Savage, 2019; Shen et al., 2019). Cas12a is a type V CRISPR-effector protein with greater specificity for genome editing in mammals and plants (Fagerlund et al., 2015; Zetsche et al., 2015). To overcome the limitation of Cas9 for antiviral research in *B. mori*, we engineered an improved CRISPR/Cas12a system and evaluated its efficiency and accuracy for BmNPV genome editing. This application expands the use of CRISPR technology in insect populations.

RNase activity of AsCas12a, FnCas12a, and LbCas12a has been used for genome editing (Fonfara et al., 2016; Kleinstiver et al., 2016; Mahfouz, 2017). AsCas12a has been previously known to efficiently edit insect genomes. However, it was not known which Cas12a system has higher editing efficiency in the Lepidoptera species, such as *B. mori*. Therefore, we compared the ability of AsCas12a, FnCas12a, and LbCas12a to edit genomes in BmN-SWU1 cells, and demonstrated that the FnCas12a system has potential for use in the development of virus-resistant silkworm lines.

Cas9 system transgenic positive lines can fully edit the target gene (Dong et al., 2018a). To determine the reason for the difference in the antiviral abilities of the Cas9 and Cas12a systems, we constructed transgenic lines. The transgenic FnCas12a × gIE1 lines could create larger fragment deletions compared with SpCas9 × sgIE1 lines. Based on the gene-editing principles of the Cas9 and Cas12a systems, we believe that the cleavage site was distant from the target site of the Cas12a system, and the target site was not destroyed after cleavage (Fagerlund et al., 2015). After the target is cut, the double chain will break, resulting in the deletion of large fragments (Mahfouz, 2017). It also had a greater impact on the function of the viral gene, which could inhibit viral DNA replication. In contrast, the Cas9 system produced blunt ends after editing, and was easily repaired by homologous recombination. The cleavage site of Cas9 was at the target site, resulting in the failure of the system to recognize it again.

Silkworm selection for virus resistance is a traditional method used in the sericulture industry. Interfering with the key genes of BmNPV or overexpression of resistance genes can increase the antiviral ability of the silkworm (Isobe et al., 2004; Subbaiah et al., 2013; Wang et al., 2013). The CRISPR/Cas9 gene-editing system has allowed us to improve the antiviral ability of transgenic silkworms. This is accomplished by editing the virus early transcriptional activators, editing multiple target sites and genes editing, and editing host-dependent factors (Smith and Johnson, 1988; Dong et al., 2018b). Increased antiviral ability, using traditional means, has reached a limit, and new technology is needed further to increase the resistance to virus attack. We used three different Cas12a systems for editing the BmNPV genome, and screened a Cas12a system with high antiviral ability and gene-editing efficiency in *B. mori*. This research demonstrated that the antiviral ability of the Cas12a system can be improved compared with the Cas9 system under the same target site in transgenic silkworms (Figure 4A). The Cas12a system can drive many crRNAs through a U6 promoter. In further research we can edit the BmNPV genome through multiple genes and multiple target sites. This will increase the negative effects on the BmNPV genome and improve the virus resistance of transgenic silkworms. We can also try to edit multiple silkworm viruses by synthesizing more crRNAs (such as crRNAs of *B. mori* densovirus, *B.mori* cytoplasmic polyhedrosis virus and other infectious diseases of *B. mori*) to one vector. This will further expand the scope and efficiency of transgenic antiviral breeding.

## Conclusion

We developed a novel CRISPR nuclease platform, AsCas12a, FnCas12a, and LbCas12a, which can be used for BmNPV genome editing and breeding of virus-resistant silkworms. Our research data indicated that the CRISPR/Cas12a system is a powerful tool for silkworm selection. The system can be used to improve silkworm virus resistance and also as a way to combat other infectious diseases. The successful application of the CRISPR/Cas12a genome editing system can be used to address diseases in *B. mori* and perhaps other economically important insects.

## Materials and Methods

### Cells

A *B. mori* cell line BmN-SWU1, derived from ovary tissue, was maintained in our laboratory and used in this study (Pan et al., 2010). BmN-SWU1 cell lines were cultured at 27°C in TC-100 medium (United States Biological, United States). The medium was supplemented with 10% (V/V) fetal bovine serum (FBS) (Gibco, United States).

### Viruses

A recombinant BmNPV (vA4^prm^-EGFP) was constructed and used in this study (Zhang et al., 2014). The baculovirus contained a gene encoding for an EGFP marker gene under the control of the *B. mori* actin A4 promoter. Budded virus (BV) amplification was performed by infection with BmN-SWU1, and then the BV was harvested at 120 h p.i. Viral titration was performed using the plaque assay method. Occlusion-derived virus (ODV) amplification was performed using oral inoculation with the wild-type (WT) Chongqing strain of BmNPV in silkworm larvae. OBs were harvested from the infected hemolymph before the larvae died (Dong et al., 2014).

### Silkworm Strains

The control (normal silkworm strain) and transgenic Cas9 strain of *B. mori* were maintained in our laboratory (Moon et al., 2019). Silkworm larvae were fed on fresh mulberry leaves and maintained at 25°C under standard conditions.

### Vector Construction

To explore whether the CRISPR/Cas12a system could be used for gene-editing in *B. mori*, wild-type (WT) LbCas12a plasmid; pY016 (pcDNA3.1-LbCas12a, Addgene plasmid # 69988), AsCas12a plasmid; pY010 (pcDNA3.1-AsCas12a, Addgene plasmid # 69982); and FnCas12a plasmid, pY004 (pcDNA3.1-FnCas12a, Addgene plasmid # 69976), were obtained from Addgene (Watertown, MA, United States). AsCas12a, FnCas12a, and LbCas12a fragments were cloned into the pSL1180-IE2^prm^-OpIE2-PA vector by digestion with *BamH I* and *Kpn I* restriction sites, yielding pSL1180-OpIE2^prm^-AsCas12a-OpIE2-PA, pSL1180-IE2^prm^-FnCas12a-OpIE2-PA, and pSL1180-OpIE2^prm^-LbCas12a-OpIE2-PA. The crRNA expression cassette under the control of the *B. mori* U6 promoter was synthesized by BGI and named pSL1180-U6-gRNA. The candidate crRNA target sequences were designed using CRISPR design software^1^. We sequentially linked the U6-gRNA expression cassettes into the pSL1180-OpIE2^prm^-AsCas12a-OpIE2-PA, pSL1180-OpIE2^prm^-FnCas12a-OpIE2-PA, and pSL1180-OpIE2^prm^-LbCas12a-OpIE2-PA, and then used restriction enzymes to verify cloning, Cas9 and sgRNA expression cassettes of the target gene *ie-1* used previous constructs. We selected the target sites of the BmNPV *ie-1* gene as CRISPR/Cas12a and CRISPR/Cas9 gene-editing sites. Sequences for all of the targets of the guide RNAs are provided in Supplementary Table S1.

The transgenic silkworm Cas9 lines were constructed as previously reported. To obtain the green fluorescent protein transgenic vector pBac [OpIE2prm-FnCas12a-OpIE2-PA-3 × P3 EGFP afm], the fragment OpIE2prm-FnCas12a-OPIE2-PA was ligated to the pBac [3 × P3 EGFP afm] vector after a single digestion of pSL1180-OpIE2prm-FnCas12a-OpIE2-PA by *Asc I* restriction endonuclease. Simultaneously, *ie-1* target gene vectors pSL1180-U6-gIE1 and pSL1180-U6-sgIE1 were ligated to a pBac [3 × P3 DsRed afm] vector after single digestion with *Bgl II*, which generated a red fluorescent protein transgenic vector for pBac [U6-gIE1 DsRed afm] and pBac [U6-sgIE1-3 × P3 DsRed afm]. All of the primers used are listed in Supplementary Table S1, and all of the constructed vectors were verified by sequencing.

### sgRNA and gRNA Design

BmNPV *ie-1* genes were used as targets for gene-editing. To avoid the influence of target sites on the editing efficiency of different gene-editing systems, we chose the same site of *ie-1* (located at 360 transcription start site of *ie-1*) as the target site of the Cas12a and Cas9 gene-editing system. We predicted sgIE1 target gene sequences using an online analysis tool^2^ (Naito et al., 2015). All of the candidate sgRNA target sequences have the GN19NGG sequence. The candidate gRNA target sequences were designed using a CRISPR design software tool^1^ (Stemmer et al., 2015). All of the candidate gRNA target sequences met the requirements of the TTTN PAM recognition domain.

### qPCR DNA Replication Assay

Total DNA was extracted from silkworm cells and larvae using a Wizard Genomic DNA extraction kit (Promega, United States). The copy number of BmNPV was calculated based on quantitative PCR as previously described (Dong et al., 2014). PCR was performed in a 15 μL reaction mixture containing using 1 μL of extracted DNA solution as the template. All of the experiments were repeated three times.

### Western Blot Analysis

After BmN-SWU1 cells were transfected with the indicated plasmids, the cellular protein was extracted in IP buffer containing 10 μL protease inhibitors (PMSF) and boiled for 10 min. Protein suspension samples were separated by 12% SDS-PAGE and then transferred to a nitrocellulose membrane. The membrane was incubated with mouse α-HA (1:2,000; Abcam, United Kingdom), mouse α-PCNA (1:2000; Abcam, United Kingdom), rabbit α-Tubulin (1:5000; Sigma, United States) and rabbit α-VP39 (1:2000) for 1 h. Then, the membrane was further incubated with HRP-labeled goat anti-mouse IgG (1:2000; Beyotime, China) and HRP-labeled goat anti-rabbit IgG (1:2000; Beyotime) for 1 h. Finally, the signals on the membrane were visualized by Clarity Western ECL Substrate (Bio-Rad, United States) following the manufacturer’s instructions. Tubulins were used to estimate the total protein levels.

### Mutagenesis Analysis at Target Sites

The purified BmNPV genome DNA products were amplified by PCR, and the resulting products were ligated into a pEASY-T5 Zero cloning vector (TransGen Biotech, China). The plasmid was analyzed by Sanger sequencing using M13 primers and aligned with the *ie-1* sequence. All of the primers used for detection are presented in Supplementary Table S1.

### Microinjection and Screening

The transgenic vectors pBac [OpIE2prm-FnCas12a-OpIE2-PA-3 × P3 EGFP afm], pBac [U6-gIE1-3 × P3 DsRed afm], and pBac [U6-sgIE1-3 × P3 DsRed afm] were mixed with the helper plasmid pHA3PIG in the ratio of 1:1 and injected into silkworm eggs as previously described (Dong et al., 2018a). The positive individuals were screened by fluorescence microscopy. Double-positive individuals FnCas12a × gIE1 were obtained by crossing FnCas12a and gIE1, and double-positive individuals SpCas9 × sgIE1 were obtained by crossing SpCas9 and sgIE1. All of the positive strains were identified by PCR amplification and fluorescence screening.

### Off-Target Assays

Potential off-target sites in the silkworm genome were predicted using CRISPR design software^2^ (Naito et al., 2015). We screened three potential sites of gIE1 with the highest off-target efficiency and examined them by PCR amplification. The corresponding PCR products were sequenced, and then aligned with the IE1 sequence. All of the off-target site primers used in the study are presented in Supplementary Table S1.

### Mortality Analyses

The OBs of BmNPV were purified from diseased larvae and stored at 4°C. The transgenic silkworms FnCas12a × gIE1 and SpCas9 × sgIE1 were inoculated with 1 × 10^6^ OBs/larva during the fourth instar. Each experimental group contained 30 larvae, and the test was performed in triplicate. Each experimental group was reared individually, and we calculated the survival rate 10 days post-inoculation.

### Determination of Expression Levels by RT-PCR

Total RNA was isolated from each cell or leave, and the cDNAs were synthesized using a cDNA synthesis kit (OMEGA, United States). Gene expression was determined by RT-PCR analysis using an Applied Biosystems 7500 Real-Time PCR System (Life Technologies, United States) with SYBR Select Master Mix Reagent (Bio-Rad). The housekeeping gene (*B. mori sw22934*) was used as a control. The 2^–ΔΔCT^ method was used to calculate the normalized expression of each sample, which was reported as a fold change (Pfaffl, 2001). Three replicates were performed for each reaction. The RT-PCR specific primers are listed in Supplementary Table S1.

### Characteristics Analysis of Transgenic Silkworm

The cocoon volumes of the two transgenic lines, FnCas12a × gIE1 and SpCas9 × sgIE1, were analyzed after pupation. Each transgenic line, including 30 larvae, was characterized by the mean of three independent replicates. The cocoon shell rate was calculated as the combined pupa and cocoon weight.

### Statistical Analysis

All of the data are expressed as mean ± SD of three independent experiments. Statistical analyses were performed with a two-sample Student’s *t-*test using GraphPad Prism 6. Differences were considered highly significant at *P* < 0.01.

## Data Availability Statement

All datasets generated for this study are included in the article/Supplementary Material.

## Author Contributions

ZD, QQ, and LH performed the vector cloning, sequencing, cell cultures, and PCR. ZD, QQ, and XZ performed the transgenic injection. JM and ZH performed the mortality analyses and DNA replication assay. ZD, MP, and CL conceived the experimental design and helped with data analysis. ZD, MP, PC, and CL prepared the manuscript. The final manuscript was reviewed and approved by all authors.

## Conflict of Interest

The authors declare that the research was conducted in the absence of any commercial or financial relationships that could be construed as a potential conflict of interest.

## Footnotes

1 https://crispr.cos.uni-heidelberg.de/index.html

2 http://crispr.dbcls.jp/

## Supplementary Material

TABLE S1 Sequences of the primers used in this study.
