# Supplementary material for: CRISPR/Cas12a Mediated Genome Editing Enhances Bombyx mori Resistance to BmNPV
Source: Front Bioeng Biotechnol. 2020 Jul 15;8:841. doi: 10.3389/fbioe.2020.00841 (PMC7373793; doi:10.3389/fbioe.2020.00841)
Supplement: TABLE S1 — Sequences of the primers used in this study. [file Table_1.DOCX]

**Supplementary Table 1. Sequences of primers used in this study.**

| Primer name | Primer sequence^*^ |
| --- | --- |
| CRISPR/Cas12a system primers | |
| U6-Bgl II/F | 5' GAAGATCTAGGTTATGTAGTACACATT 3' |
| gIE360-Bgl II /R  (AsCpf1) | 5'GAAGATCTAAAAAATGGTTCACACCAACAGACTGCTCATCTACAAGAGTAGAAATTACACTTGTAGAGCACGATATTTTGT3' |
| gIE360-Bgl II /R  (FnCpf1) | 5'GAAGATCTAAAAAATGGTTCACACCAACAGACTGCTCATCTACAACAGTAGAAATTACACTTGTAGAGCACGATATTTTGT3' |
| gIE360-Bgl II /R  (LbCpf1) | 5'GAAGATCTAAAAAATGGTTCACACCAACAGACTGCTCATCTACACTTAGTAGAAATTACACTTGTAGAGCACGATATTTTGT3' |
| CRISPR/Cas9 system primers | |
| U6-Bgl II/F | 5' GAAGATCTAGGTTATGTAGTACACATT 3' |
| U6-Bgl II/R | 5' TTAGATCTAAAAAAAGCACCGACTCG 3' |
| sgIE1-352/F | 5' AAGTGAATCTTTTGAGCAGTCTGT 3' |
| sgIE1-352/R | 5' AAACACAGACTGCTCAAAAGATTC 3' |
| RT-PCR primers | |
| RT-IE1/F | 5' CGAGACGGCTGCACAAAA 3' |
| RT-IE1/R | 5' TGCCCAAAAGAAACCCACA 3' |
| RT-VP39/F | 5' AGACACCACAAACCCGAACAC 3' |
| RT-VP39/R | 5' TTGATCGCCAACACCACCT 3' |
| RT-Poly/F | 5' GCAGTGTGAAACCCGATACCAT 3' |
| RT-Poly/R | 5' CCACCTAAGAGCGTGTTGAGC 3' |
| RT-GP64/F | 5' CACCATCGTGGAGACGGACTAC 3' |
| RT-GP64/R | 5' ACCTCGCACTGCTGCCTGA 3' |
| RT-sw22934/F | 5' TTCGTACTGCTCTTCTCGT 3' |
| RT-sw22934/R | 5' CAAAGTTGATAGCAATTCCCT 3' |
| GP41 primers | |
| GP41/F | 5' CCTATTCTGTGCTGGTGGTGG 3' |
| GP41/R | 5' ATGTTGATGTGCGGAAAGC 3' |
| Detection primers | |
| IE1-F1 | 5' ATGACGCAAATTAATTTTAAC 3' |
| IE1-R640 | 5' GGTCGGAGAACCTGTTGGAA 3' |
| IE1-R1208 | 5' GGGGATTGTCGGGAAA 3' |
| Off-target primers | |
| OT1-gIE360F | 5' CGAAGAGCACTCGTGACAATAAAA 3' |
| OT1-gIE1360R | 5' CGTTTCAAAAAGAACACATAATAGC 3' |
| OT2-gIE1360F | 5' AGTTAAGGTTCCGCTGAAATAGTCT 3' |
| OT2-gIE1360R | 5' TTTTTACCCAGTTCAGCATATTGAG 3' |
| OT3-gIE1360F | 5' ACAGCAGCAAGTCTGACTACTGTTG 3' |
| OT1-gIE1360F | 5' CGATCGTGTTGACTTGATTTGTAAA 3' |

^*^ (The restriction enzyme sites are marked in red, crRNA sequence is marked in blue.)
